# Decommissioning care: The need for rigorous multifaceted evaluations of decisions to withdraw health services

**DOI:** 10.1371/journal.pmed.1002413

**Published:** 2017-10-31

**Authors:** Aziz Sheikh

**Affiliations:** Usher Institute of Population Health Sciences and Informatics, The University of Edinburgh, Edinburgh, United Kingdom

## Abstract

In this Perspective on the two clinical trials of Terry Haines and colleagues that incrementally removed and reinstated allied healthcare services, Aziz Sheikh discusses the evidence base for the routine provision of such services.

In the current issue of *PLOS Medicine*, Terry Haines and colleagues report on 2 trials undertaken in acute medical and surgical wards in Australia to study the highly topical question of whether withdrawing weekend allied health services leads to detrimental health outcomes [1]. Their trials offer a clear answer to an important health policy question and, more importantly, also provide the first published example of the use of a novel “incremental removal and reinstatement” cluster randomised, noninferiority, stepped-wedge design that can help reframe deliberations on disinvestment of care. Instead of focusing on potential cost-cutting measures, these trials offer a more nuanced assessment of value-based care whilst simultaneously providing a rigorous, unbiased assessment of the impact of radical restructuring of healthcare provision.

## Increasing policy interest in reducing unnecessary care

There is growing global policy interest in reducing unnecessary care, motivated by the recognition that overprovision of healthcare is a major contributor to the substantial iatrogenic harm experienced by patients and that this is also responsible for considerable unnecessary healthcare expenditure [2,3,4]. With health systems being financially challenged by the combination of increasing numbers of people living with long-term conditions and government restraints on healthcare expenditure, removing care that does not add value is an obvious policy target. Much of the drive to remove unnecessary care has, however, been focused on care at the level of the individual patient—for example, reducing investigations such as magnetic resonance imaging (MRI) of the lumbar spine in simple mechanical back pain and avoiding prescriptions of unnecessary medications such as antibiotics for viral upper respiratory tract infections [5,6]. The development of clinical practice guidelines that make clear the evidence base underpinning recommendations for care has been important in this respect, and it is encouraging to see that these, particularly when combined with computerised decision support tools and change-management initiatives, can translate into greater provision of evidence-based care [7].

## Investigating the decommissioning of care

Whilst such rationalising of individual patient care is of undoubted clinical value, far more important from a health policy perspective are decisions to withdraw services that are not adding value. This is, however, fraught with challenges, as decisions to withdraw services are often interpreted as politically and/or profit-motivated, cost-saving measures. This is perhaps best illustrated by considering the public (and often also professional) outcry associated with attempts to close down local hospitals in the context of restructuring health services to focus more on community-based, primary care provision. Such politically explosive decisions are, as a result, often deferred or aborted, with the consequence that potentially unnecessary, expensive care provision continues [8].

The study by Haines and colleagues sought formally to study the impact of a decision to withdraw weekend allied health services—this including dietetics, speech therapy, occupational therapy, and physiotherapy—from acute medical and surgical wards in 2 Australian hospitals [1]. Building on their earlier theoretical work on designs to study decommissioning of care [9], they undertook 2 elegant cluster randomised stepped-wedge trials that allowed them to study the effects of withdrawing weekend allied health services and then to investigate the reintroduction of more contextually tailored versions of these services to the same hospital wards. A key advantage of their design is that the commitment to incrementally reinstating the service in question can help quell some of the inevitable public and professional concerns that surround deliberations about incrementally withdrawing historically embedded service provision. Importantly, it also provides a much more rigorous and unbiased assessment of the impact of the intervention than is possible using quality improvement approaches, thereby allowing generalisable inferences beyond the immediate acute care settings in which these trials were undertaken.

The decision to undertake parallel qualitative, process, and health economic evaluation is also welcome. Once reported, these will yield valuable additional insights into stakeholder perceptions, causal mechanisms, and the economic implications of this decision to disinvest in weekend allied health services, all of which will help to inform decisions on the appropriateness of implementing such disinvestments in other care settings [10].

## Wider application of the “incremental removal and reinstatement” design

This is, as far as I’m aware, one of the first uses of cluster stepped-wedge trials to study decisions of disinvesting in care provision and the first published example of this novel “incremental removal and reinstatement” trial design. This approach has the potential to be extended to a whole range of other services that are currently delivered as routine care but that have a questionable underpinning evidence base. Examples include routine child-development assessments and annual general health checks. Systematic comparisons of differences in care provision between health systems internationally will help to highlight numerous other examples of care that should similarly be considered for formal disinvestment decisions [11]. Now that proof of principle of this novel health services research design has been established, consideration should also be given to undertaking hybrid effectiveness-implementation disinvestment designs, which can help support the timely translation of findings from such disinvestment trials into routine provision of care [12].
